# 3,3′-Di-2-naphthoyl-1,1′-(*o*-phenyl­ene)dithio­urea

**DOI:** 10.1107/S1600536808026299

**Published:** 2008-08-20

**Authors:** Hai-Tang Du, Hai-Jun Du, Weiyi Zhou

**Affiliations:** a Institute of Natural Products, Research Center for Eco-Environmental Sciences, Guiyang College, Guiyang 550005, People’s Republic of China; b School of Chemistry and Environmental Sciences, Guizhou University for Nationalities, Guiyang 550025, People’s Republic of China; c Analytical Center, Tianjin University, Tianjin 300072, People’s Republic of China

## Abstract

In the mol­ecule of the title compound, C_30_H_22_N_4_O_2_S_2_, the central benzene ring is oriented at dihedral angles of 63.83 (3) and 1.37 (3)° with respect to the naphthalene ring systems, while the two naphthalene ring systems are oriented at a dihedral angle of 62.78 (3)°. Intra­molecular N—H⋯O and N—H⋯N hydrogen bonds result in the formation of one five- and two six-membered rings. The twisting modes of the two side arms are different [C—N—C—O and C—N—C—N torsion angles = 11.1 (4) and 1.5 (3)°, respectively, in one arm, and −2.2 (4) and 0.8 (3)° in the other arm]. In the crystal structure, inter­molecular N—H⋯S hydrogen bonds link the mol­ecules into centrosymmetric dimers. There is a C—H⋯π contact between the naphthalene rings and π–π contacts between the naphthalene rings and the naphthalene and benzene rings [centroid–centroid distances = 3.651 (1), 3.828 (1), 3.811 (2) and 3.786 (1) Å].

## Related literature

For a related structure, see: Thiam *et al.* (2008 ▶). For ring conformation puckering parameters, see: Cremer & Pople (1975 ▶).
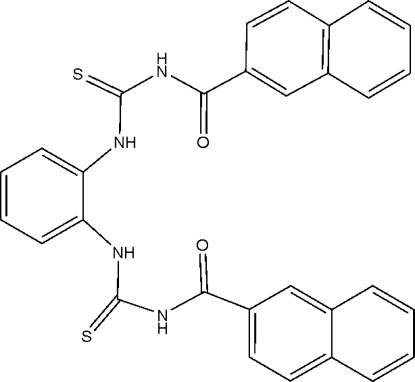


## Experimental

### 

#### Crystal data

C_30_H_22_N_4_O_2_S_2_
*M* *_r_* = 534.64
Triclinic, 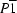

*a* = 8.7135 (17) Å
*b* = 12.453 (3) Å
*c* = 12.541 (3) Å
α = 72.33 (3)°
β = 74.55 (3)°
γ = 78.89 (3)°
*V* = 1240.5 (6) Å^3^
*Z* = 2
Mo *K*α radiation
μ = 0.25 mm^−1^
*T* = 113 (2) K
0.10 × 0.08 × 0.04 mm

#### Data collection

Rigaku Saturn CCD area-detector diffractometer
Absorption correction: multi-scan (*CrystalClear*; Rigaku/MSC, 2005 ▶) *T*_min_ = 0.975, *T*_max_ = 0.990
7203 measured reflections
4337 independent reflections
3311 reflections with *I* > 2σ(*I*)
*R*_int_ = 0.055

#### Refinement

*R*[*F* ^2^ > 2σ(*F* ^2^)] = 0.050
*wR*(*F* ^2^) = 0.130
*S* = 1.07
4337 reflections
355 parameters
H atoms treated by a mixture of independent and constrained refinement
Δρ_max_ = 0.32 e Å^−3^
Δρ_min_ = −0.38 e Å^−3^

### 

Data collection: *CrystalClear* (Rigaku/MSC, 2005 ▶); cell refinement: *CrystalClear*; data reduction: *CrystalStructure* (Rigaku/MSC, 2005 ▶); program(s) used to solve structure: *SHELXS97* (Sheldrick, 2008 ▶); program(s) used to refine structure: *SHELXL97* (Sheldrick, 2008 ▶); molecular graphics: *SHELXTL* (Sheldrick, 2008 ▶); software used to prepare material for publication: *SHELXTL*.

## Supplementary Material

Crystal structure: contains datablocks I, global. DOI: 10.1107/S1600536808026299/hk2511sup1.cif

Structure factors: contains datablocks I. DOI: 10.1107/S1600536808026299/hk2511Isup2.hkl

Additional supplementary materials:  crystallographic information; 3D view; checkCIF report

## Figures and Tables

**Table 1:** Hydrogen-bond geometry (Å, °)

*D*—H⋯*A*	*D*—H	H⋯*A*	*D*⋯*A*	*D*—H⋯*A*
N1—H1⋯O1	0.89 (2)	1.88 (3)	2.624 (3)	140 (2)
N2—H2*A*⋯S1^i^	0.82 (3)	2.60 (3)	3.418 (2)	178 (2)
N3—H3*A*⋯O2	0.83 (3)	1.88 (3)	2.613 (3)	148 (3)
N3—H3*A*⋯N1	0.83 (3)	2.28 (3)	2.693 (3)	111 (2)
C28—H28⋯*Cg*3	0.95	2.76	3.621 (2)	152 (2)

Symmetry code: (i) 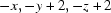. *Cg*3 is the centroid of the C11–C16 ring.

**Fig. 1.**
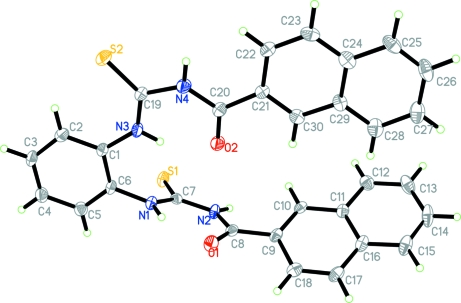
The molecular structure of the title molecule, with the atom-numbering scheme. Displacement ellipsoids are drawn at the 50% probability level.

**Fig. 2.**
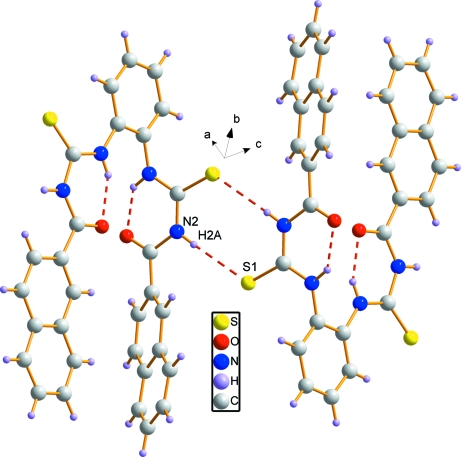
A partial packing diagram of the title compound. Hydrogen bonds are shown as dashed lines.

## supplementary crystallographic information

### Comment

In the molecule of the title compound (Fig. 1), the bond lengths and angles are within normal ranges. Rings A (C1-C6), B (C9-C11/C16-C18), C (C11-C16), D (C21-C24/C29/C30) and E (C24-C29) are, of course, planar, and the dihedral angles between rings B, C and D, E are B/C = 4.38 (4)° and D/E = 3.00 (3)°. So, the naphthalene rings are nearly planar, and the dihedral angle between them is 62.78 (3)°. Ring A is oriented with respect to the naphthalene rings, consisting of B, C and D, E rings, at dihedral angles of 63.83 (3)° and 1.37 (3)°, respectively.

The intramolecular N-H···O and N-H···N hydrogen bonds (Table 1) result in the formation of one five- and two six-membered rings: F (N1/N3/C1/C6/H3A), G (O1/N1/N2/C7/C8/H1) and H (O2/N3/N4/C19/C20/H3A). Rings F and H are planar and they are oriented at a dihedral angle of 6.25 (3)°. Ring A is oriented with respect to them at dihedral angles of 1.25 (3)° and 5.48 (3)°, respectively. Ring G adopts flattened-boat [φ = -71.32 (2)°, θ = 60.93 (3)°] conformation, having total puckering amplitude, Q_T_, of 0.371 (3) Å (Cremer & Pople, 1975). The two side arms are not twisted in the same way, as evidenced by the torsion angles: C7-N2-C8-O1 [11.1 (4)°], C8-N2-C7-N1 [1.5 (3)°] and C19-N4-C20-O2 [-2.2 (4)°], C20-N4-C19-N3 [0.8 (3)°], as in 1,2-bis(N'-benzoyl- thioureido)benzene (Thiam *et al.*,  2008).

In the crystal structure, intermolecular N-H···S hydrogen bonds (Table 1) link the molecules into centrosymmetric dimers (Fig. 2), in which they may be effective in the stabilization of the structure. The C—H···π contact (Table 1) between the naphthalene rings and the π—π contacts between the naphthalene rings and the naphthalene and phenyl rings: Cg4···Cg4^i^, Cg2···Cg3^ii^, Cg3···Cg3^i^and Cg5···Cg1^iii^ [symmetry codes: (i) 2 - x, -y, 1 - z; (ii) 2 - x, 1 - y, -z; (iii) 1 - x, -y, 1 - z, where Cg1, Cg2, Cg3, Cg4 and Cg5 are centroids of the rings A, B, C, D and E, respectively] further stabilize the structure, with centroid-centroid distances of 3.651 (1), 3.828 (1), 3.811 (2) and 3.786 (1) Å, respectively.

### Experimental

For the preparation of the title compound, ammonium thiocyanate (30 mmol), 2-naphthoyl chloride (20 mmol), PEG-400 (0.2 mmol) and acetone (50 mL) were placed in a dried round-bottomed flask containing a magnetic stirrer bar and stirred at room temperature for 1 h, then benzene-1,2-diamine (9.5 mmol) was added, and the mixture was stirred for 2 h. The mixture was poured into water (20 ml). The resulting solid was filtered, washed with water, and then dried. Crystals suitable for X-ray analysis were obtained by the recrystallization of the solid residue from a mixture of N,N-dimethylformamide/ethanol (1:1) by slow evaporation at room temperature.

### Refinement

H1, H2A, H3A, H4A (for NH) atoms were located in difference syntheses and refined [N-H = 0.82 (3)-0.89 (2) Å and U_iso_(H) = 0.021-0.027 Å^2^]. The remaining H atoms were positioned geometrically, with C-H = 0.95 Å for aromatic H and constrained to ride on their parent atoms with U_iso_(H) = 1.2U_eq_(C).

### Crystal data

**Table:**

C_30_H_22_N_4_O_2_S_2_	*Z* = 2
*M**_r_* = 534.64	*F*_000_ = 556
Triclinic, *P*1	*D*_x_ = 1.431 Mg m^−^^3^
Hall symbol: -P 1	Melting point: 489 K
*a* = 8.7135 (17) Å	Mo *K*α radiation λ = 0.71073 Å
*b* = 12.453 (3) Å	Cell parameters from 2566 reflections
*c* = 12.541 (3) Å	θ = 1.7–27.1º
α = 72.33 (3)º	µ = 0.25 mm^−^^1^
β = 74.55 (3)º	*T* = 113 (2) K
γ = 78.89 (3)º	Block, colorless
*V* = 1240.5 (6) Å^3^	0.10 × 0.08 × 0.04 mm

### Data collection

**Table:**

Rigaku Saturn CCD area-detector diffractometer	4337 independent reflections
Radiation source: rotating anode	3311 reflections with *I* > 2σ(*I*)
Monochromator: confocal	*R*_int_ = 0.055
Detector resolution: 7.31 pixels mm^-1^	θ_max_ = 25.0º
*T* = 113(2) K	θ_min_ = 1.7º
ω and φ scans	*h* = −9→10
Absorption correction: multi-scan(CrystalClear; Rigaku/MSC, 2005)	*k* = −14→14
*T*_min_ = 0.975, *T*_max_ = 0.990	*l* = −14→12
7203 measured reflections	

### Refinement

**Table:**

Refinement on *F*^2^	Secondary atom site location: difference Fourier map
Least-squares matrix: full	Hydrogen site location: inferred from neighbouring sites
*R*[*F*^2^ > 2σ(*F*^2^)] = 0.050	H atoms treated by a mixture of independent and constrained refinement
*wR*(*F*^2^) = 0.130	*w* = 1/[σ^2^(*F*_o_^2^) + (0.0613*P*)^2^] where *P* = (*F*_o_^2^ + 2*F*_c_^2^)/3
*S* = 1.07	(Δ/σ)_max_ = 0.001
4337 reflections	Δρ_max_ = 0.32 e Å^−^^3^
355 parameters	Δρ_min_ = −0.38 e Å^−^^3^
Primary atom site location: structure-invariant direct methods	Extinction correction: none

### Special details

**Table:**

Geometry. All e.s.d.'s (except the e.s.d. in the dihedral angle between two l.s. planes) are estimated using the full covariance matrix. The cell e.s.d.'s are taken into account individually in the estimation of e.s.d.'s in distances, angles and torsion angles; correlations between e.s.d.'s in cell parameters are only used when they are defined by crystal symmetry. An approximate (isotropic) treatment of cell e.s.d.'s is used for estimating e.s.d.'s involving l.s. planes.
Refinement. Refinement of F^2^ against ALL reflections. The weighted R-factor wR and goodness of fit S are based on F^2^, conventional R-factors R are based on F, with F set to zero for negative F^2^. The threshold expression of F^2^ > 2sigma(F^2^) is used only for calculating R-factors(gt) etc. and is not relevant to the choice of reflections for refinement. R-factors based on F^2^ are statistically about twice as large as those based on F, and R- factors based on ALL data will be even larger.

### Fractional atomic coordinates and isotropic or equivalent isotropic displacement parameters (Å^2^)

**Table:**

	*x*	*y*	*z*	*U*_iso_*/*U*_eq_	
S1	0.14334 (7)	1.12794 (6)	0.94643 (6)	0.02210 (19)	
S2	0.47504 (8)	1.33865 (7)	0.40927 (6)	0.0302 (2)	
O1	0.48084 (19)	0.83813 (16)	0.82983 (16)	0.0242 (4)	
O2	0.30123 (19)	1.00328 (16)	0.63446 (14)	0.0231 (4)	
N1	0.4251 (2)	1.05227 (19)	0.83207 (17)	0.0178 (5)	
H1	0.488 (3)	0.992 (2)	0.814 (2)	0.021*	
N2	0.2372 (2)	0.92516 (18)	0.90634 (17)	0.0179 (5)	
H2A	0.146 (3)	0.911 (2)	0.941 (2)	0.021*	
N3	0.4218 (2)	1.1856 (2)	0.61854 (19)	0.0199 (5)	
H3A	0.384 (3)	1.124 (3)	0.650 (3)	0.024*	
N4	0.3551 (2)	1.1461 (2)	0.47074 (19)	0.0223 (5)	
H4A	0.356 (3)	1.171 (3)	0.397 (3)	0.027*	
C1	0.4825 (2)	1.2304 (2)	0.6873 (2)	0.0173 (5)	
C2	0.5430 (3)	1.3339 (2)	0.6541 (2)	0.0219 (6)	
H2	0.5435	1.3827	0.5792	0.026*	
C3	0.6027 (3)	1.3653 (2)	0.7308 (2)	0.0219 (6)	
H3	0.6460	1.4353	0.7075	0.026*	
C4	0.6001 (3)	1.2962 (2)	0.8410 (2)	0.0240 (6)	
H4	0.6407	1.3192	0.8927	0.029*	
C5	0.5384 (3)	1.1938 (2)	0.8755 (2)	0.0221 (6)	
H5	0.5356	1.1465	0.9512	0.026*	
C6	0.4806 (3)	1.1607 (2)	0.7992 (2)	0.0172 (5)	
C7	0.2774 (3)	1.0316 (2)	0.8916 (2)	0.0168 (5)	
C8	0.3352 (3)	0.8358 (2)	0.8680 (2)	0.0178 (5)	
C9	0.2575 (3)	0.7382 (2)	0.8759 (2)	0.0181 (5)	
C10	0.1037 (3)	0.7483 (2)	0.86101 (19)	0.0175 (5)	
H10	0.0395	0.8199	0.8535	0.021*	
C11	0.0400 (3)	0.6543 (2)	0.8567 (2)	0.0187 (5)	
C12	−0.1169 (3)	0.6634 (2)	0.8383 (2)	0.0252 (6)	
H12	−0.1859	0.7327	0.8362	0.030*	
C13	−0.1691 (3)	0.5734 (3)	0.8237 (2)	0.0302 (7)	
H13	−0.2731	0.5810	0.8098	0.036*	
C14	−0.0691 (3)	0.4691 (3)	0.8292 (2)	0.0317 (7)	
H14	−0.1058	0.4072	0.8179	0.038*	
C15	0.0794 (3)	0.4564 (2)	0.8507 (2)	0.0286 (7)	
H15	0.1444	0.3852	0.8560	0.034*	
C16	0.1376 (3)	0.5477 (2)	0.8651 (2)	0.0213 (6)	
C17	0.2939 (3)	0.5386 (2)	0.8852 (2)	0.0228 (6)	
H17	0.3583	0.4670	0.8957	0.027*	
C18	0.3520 (3)	0.6298 (2)	0.8896 (2)	0.0217 (6)	
H18	0.4571	0.6217	0.9019	0.026*	
C19	0.4172 (3)	1.2218 (2)	0.5070 (2)	0.0191 (6)	
C20	0.2990 (3)	1.0445 (2)	0.5323 (2)	0.0198 (6)	
C21	0.2338 (3)	0.9850 (2)	0.4698 (2)	0.0182 (6)	
C22	0.2170 (3)	1.0319 (2)	0.3545 (2)	0.0214 (6)	
H22	0.2506	1.1039	0.3120	0.026*	
C23	0.1522 (3)	0.9726 (2)	0.3048 (2)	0.0247 (6)	
H23	0.1422	1.0042	0.2275	0.030*	
C24	0.0999 (3)	0.8658 (2)	0.3658 (2)	0.0235 (6)	
C25	0.0246 (3)	0.8052 (3)	0.3189 (2)	0.0284 (7)	
H25	0.0109	0.8356	0.2423	0.034*	
C26	−0.0282 (3)	0.7038 (3)	0.3824 (3)	0.0317 (7)	
H26	−0.0808	0.6655	0.3501	0.038*	
C27	−0.0059 (3)	0.6551 (3)	0.4952 (3)	0.0316 (7)	
H27	−0.0426	0.5841	0.5384	0.038*	
C28	0.0691 (3)	0.7109 (3)	0.5422 (2)	0.0280 (7)	
H28	0.0869	0.6770	0.6174	0.034*	
C29	0.1199 (3)	0.8177 (2)	0.4805 (2)	0.0201 (6)	
C30	0.1876 (3)	0.8804 (2)	0.5296 (2)	0.0203 (6)	
H30	0.2012	0.8490	0.6060	0.024*	

### Atomic displacement parameters (Å^2^)

**Table:**

	*U*^11^	*U*^22^	*U*^33^	*U*^12^	*U*^13^	*U*^23^
S1	0.0227 (3)	0.0185 (4)	0.0251 (3)	−0.0060 (3)	0.0016 (2)	−0.0097 (3)
S2	0.0430 (4)	0.0245 (5)	0.0217 (4)	−0.0143 (3)	−0.0095 (3)	0.0041 (3)
O1	0.0205 (9)	0.0215 (12)	0.0295 (10)	−0.0025 (7)	−0.0020 (7)	−0.0084 (9)
O2	0.0329 (10)	0.0215 (12)	0.0157 (9)	−0.0084 (8)	−0.0052 (7)	−0.0030 (9)
N1	0.0202 (10)	0.0148 (13)	0.0186 (10)	−0.0048 (9)	−0.0026 (8)	−0.0046 (10)
N2	0.0182 (9)	0.0161 (13)	0.0189 (10)	−0.0071 (9)	0.0011 (8)	−0.0053 (10)
N3	0.0232 (10)	0.0165 (14)	0.0209 (11)	−0.0080 (9)	−0.0033 (8)	−0.0042 (10)
N4	0.0297 (11)	0.0214 (14)	0.0155 (11)	−0.0066 (9)	−0.0040 (9)	−0.0031 (10)
C1	0.0167 (11)	0.0154 (15)	0.0191 (12)	−0.0041 (10)	−0.0018 (9)	−0.0039 (11)
C2	0.0220 (12)	0.0178 (16)	0.0232 (13)	−0.0021 (10)	−0.0036 (10)	−0.0029 (12)
C3	0.0199 (12)	0.0147 (16)	0.0309 (14)	−0.0049 (10)	−0.0019 (10)	−0.0072 (12)
C4	0.0238 (12)	0.0221 (17)	0.0289 (14)	−0.0046 (11)	−0.0067 (10)	−0.0091 (13)
C5	0.0231 (12)	0.0234 (17)	0.0199 (13)	−0.0059 (11)	−0.0037 (10)	−0.0051 (12)
C6	0.0157 (11)	0.0130 (15)	0.0220 (12)	−0.0036 (9)	0.0002 (9)	−0.0061 (11)
C7	0.0194 (11)	0.0193 (16)	0.0121 (11)	−0.0046 (10)	−0.0051 (9)	−0.0022 (11)
C8	0.0222 (12)	0.0156 (15)	0.0150 (11)	−0.0025 (10)	−0.0052 (9)	−0.0023 (11)
C9	0.0242 (12)	0.0149 (15)	0.0122 (11)	−0.0052 (10)	0.0012 (9)	−0.0021 (11)
C10	0.0219 (11)	0.0137 (15)	0.0143 (12)	−0.0014 (10)	−0.0022 (9)	−0.0022 (11)
C11	0.0242 (12)	0.0192 (16)	0.0116 (11)	−0.0073 (10)	−0.0002 (9)	−0.0030 (11)
C12	0.0253 (13)	0.0301 (18)	0.0209 (13)	−0.0062 (11)	−0.0044 (10)	−0.0070 (13)
C13	0.0243 (13)	0.046 (2)	0.0246 (14)	−0.0168 (12)	0.0006 (10)	−0.0136 (15)
C14	0.0381 (15)	0.035 (2)	0.0268 (15)	−0.0243 (13)	0.0089 (12)	−0.0161 (15)
C15	0.0372 (15)	0.0231 (18)	0.0237 (14)	−0.0118 (12)	0.0072 (11)	−0.0108 (13)
C16	0.0279 (13)	0.0179 (16)	0.0160 (12)	−0.0070 (10)	0.0037 (9)	−0.0062 (12)
C17	0.0255 (12)	0.0164 (16)	0.0214 (13)	0.0006 (11)	0.0019 (10)	−0.0060 (12)
C18	0.0220 (12)	0.0208 (16)	0.0186 (12)	−0.0014 (10)	−0.0009 (10)	−0.0037 (12)
C19	0.0211 (12)	0.0155 (15)	0.0201 (13)	−0.0038 (10)	−0.0033 (9)	−0.0038 (12)
C20	0.0183 (12)	0.0187 (16)	0.0202 (13)	−0.0028 (10)	−0.0007 (9)	−0.0048 (12)
C21	0.0189 (11)	0.0175 (16)	0.0162 (12)	−0.0008 (10)	−0.0011 (9)	−0.0050 (12)
C22	0.0235 (12)	0.0180 (16)	0.0198 (13)	−0.0019 (10)	−0.0014 (10)	−0.0041 (12)
C23	0.0245 (12)	0.0300 (18)	0.0188 (13)	0.0060 (11)	−0.0063 (10)	−0.0095 (13)
C24	0.0153 (11)	0.0304 (18)	0.0263 (14)	0.0049 (11)	−0.0019 (10)	−0.0166 (13)
C25	0.0225 (12)	0.041 (2)	0.0274 (14)	0.0044 (12)	−0.0082 (11)	−0.0203 (15)
C26	0.0231 (13)	0.040 (2)	0.0413 (17)	−0.0027 (12)	−0.0042 (12)	−0.0284 (17)
C27	0.0287 (14)	0.033 (2)	0.0354 (16)	−0.0093 (12)	0.0038 (12)	−0.0188 (15)
C28	0.0298 (13)	0.0314 (19)	0.0235 (14)	−0.0078 (12)	0.0014 (11)	−0.0124 (14)
C29	0.0173 (11)	0.0248 (17)	0.0191 (12)	−0.0015 (10)	0.0015 (9)	−0.0125 (12)
C30	0.0213 (12)	0.0244 (17)	0.0149 (12)	−0.0017 (10)	−0.0006 (9)	−0.0085 (12)

### Geometric parameters (Å, °)

**Table:**

S1—C7	1.677 (2)	C11—C12	1.424 (3)
S2—C19	1.657 (3)	C12—C13	1.364 (4)
O1—C8	1.234 (3)	C12—H12	0.9500
O2—C20	1.231 (3)	C13—C14	1.412 (4)
N1—C7	1.334 (3)	C13—H13	0.9500
N1—C6	1.427 (3)	C14—C15	1.362 (4)
N1—H1	0.89 (2)	C14—H14	0.9500
N2—C7	1.381 (3)	C15—C16	1.407 (4)
N2—C8	1.392 (3)	C15—H15	0.9500
N2—H2A	0.82 (3)	C16—C17	1.427 (3)
N3—C19	1.343 (3)	C17—C18	1.350 (4)
N3—C1	1.409 (3)	C17—H17	0.9500
N3—H3A	0.83 (3)	C18—H18	0.9500
N4—C20	1.372 (4)	C20—C21	1.496 (3)
N4—C19	1.405 (3)	C21—C30	1.367 (4)
N4—H4A	0.88 (3)	C21—C22	1.422 (3)
C1—C2	1.389 (4)	C22—C23	1.371 (3)
C1—C6	1.405 (4)	C22—H22	0.9500
C2—C3	1.387 (3)	C23—C24	1.412 (4)
C2—H2	0.9500	C23—H23	0.9500
C3—C4	1.385 (4)	C24—C25	1.418 (3)
C3—H3	0.9500	C24—C29	1.424 (4)
C4—C5	1.381 (4)	C25—C26	1.363 (4)
C4—H4	0.9500	C25—H25	0.9500
C5—C6	1.385 (3)	C26—C27	1.409 (4)
C5—H5	0.9500	C26—H26	0.9500
C8—C9	1.468 (3)	C27—C28	1.374 (4)
C9—C10	1.379 (3)	C27—H27	0.9500
C9—C18	1.427 (3)	C28—C29	1.409 (4)
C10—C11	1.409 (3)	C28—H28	0.9500
C10—H10	0.9500	C29—C30	1.414 (3)
C11—C16	1.423 (3)	C30—H30	0.9500
			
C7—N1—C6	123.9 (2)	C15—C14—C13	120.6 (3)
C7—N1—H1	114.4 (18)	C15—C14—H14	119.7
C6—N1—H1	121.7 (18)	C13—C14—H14	119.7
C7—N2—C8	127.1 (2)	C14—C15—C16	120.7 (3)
C7—N2—H2A	118.4 (19)	C14—C15—H15	119.7
C8—N2—H2A	114.5 (19)	C16—C15—H15	119.7
C19—N3—C1	131.8 (2)	C15—C16—C11	119.4 (2)
C19—N3—H3A	111.8 (19)	C15—C16—C17	122.4 (2)
C1—N3—H3A	116.4 (19)	C11—C16—C17	118.2 (2)
C20—N4—C19	129.6 (2)	C18—C17—C16	121.3 (2)
C20—N4—H4A	119.2 (19)	C18—C17—H17	119.4
C19—N4—H4A	111.2 (19)	C16—C17—H17	119.4
C2—C1—C6	119.0 (2)	C17—C18—C9	120.8 (2)
C2—C1—N3	126.2 (2)	C17—C18—H18	119.6
C6—C1—N3	114.8 (2)	C9—C18—H18	119.6
C3—C2—C1	119.5 (3)	N3—C19—N4	113.0 (2)
C3—C2—H2	120.2	N3—C19—S2	129.7 (2)
C1—C2—H2	120.2	N4—C19—S2	117.24 (19)
C4—C3—C2	121.1 (3)	O2—C20—N4	122.1 (2)
C4—C3—H3	119.5	O2—C20—C21	120.9 (2)
C2—C3—H3	119.5	N4—C20—C21	117.0 (2)
C5—C4—C3	120.0 (2)	C30—C21—C22	119.7 (2)
C5—C4—H4	120.0	C30—C21—C20	116.7 (2)
C3—C4—H4	120.0	C22—C21—C20	123.6 (2)
C4—C5—C6	119.5 (3)	C23—C22—C21	119.7 (3)
C4—C5—H5	120.2	C23—C22—H22	120.1
C6—C5—H5	120.2	C21—C22—H22	120.1
C5—C6—C1	120.9 (3)	C22—C23—C24	121.6 (2)
C5—C6—N1	119.9 (2)	C22—C23—H23	119.2
C1—C6—N1	119.2 (2)	C24—C23—H23	119.2
N1—C7—N2	116.73 (19)	C23—C24—C25	122.9 (3)
N1—C7—S1	123.0 (2)	C23—C24—C29	118.7 (2)
N2—C7—S1	120.30 (17)	C25—C24—C29	118.4 (3)
O1—C8—N2	121.6 (2)	C26—C25—C24	120.7 (3)
O1—C8—C9	121.5 (2)	C26—C25—H25	119.6
N2—C8—C9	116.90 (19)	C24—C25—H25	119.6
C10—C9—C18	119.0 (2)	C25—C26—C27	121.0 (2)
C10—C9—C8	123.1 (2)	C25—C26—H26	119.5
C18—C9—C8	117.6 (2)	C27—C26—H26	119.5
C9—C10—C11	121.3 (2)	C28—C27—C26	119.6 (3)
C9—C10—H10	119.4	C28—C27—H27	120.2
C11—C10—H10	119.4	C26—C27—H27	120.2
C10—C11—C16	119.3 (2)	C27—C28—C29	120.9 (3)
C10—C11—C12	122.2 (2)	C27—C28—H28	119.6
C16—C11—C12	118.4 (2)	C29—C28—H28	119.6
C13—C12—C11	120.6 (2)	C28—C29—C30	122.0 (2)
C13—C12—H12	119.7	C28—C29—C24	119.4 (2)
C11—C12—H12	119.7	C30—C29—C24	118.6 (3)
C12—C13—C14	120.3 (2)	C21—C30—C29	121.7 (2)
C12—C13—H13	119.9	C21—C30—H30	119.1
C14—C13—H13	119.9	C29—C30—H30	119.1
			
C19—N3—C1—C2	6.0 (4)	C10—C11—C16—C17	4.0 (3)
C19—N3—C1—C6	−173.4 (2)	C12—C11—C16—C17	−179.1 (2)
C6—C1—C2—C3	1.1 (3)	C15—C16—C17—C18	174.6 (2)
N3—C1—C2—C3	−178.3 (2)	C11—C16—C17—C18	−3.7 (4)
C1—C2—C3—C4	−1.3 (3)	C16—C17—C18—C9	1.0 (4)
C2—C3—C4—C5	0.5 (3)	C10—C9—C18—C17	1.6 (4)
C3—C4—C5—C6	0.5 (3)	C8—C9—C18—C17	−172.9 (2)
C4—C5—C6—C1	−0.6 (3)	C1—N3—C19—N4	175.7 (2)
C4—C5—C6—N1	176.5 (2)	C1—N3—C19—S2	−3.4 (4)
C2—C1—C6—C5	−0.2 (3)	C20—N4—C19—N3	0.8 (3)
N3—C1—C6—C5	179.30 (19)	C20—N4—C19—S2	−179.98 (18)
C2—C1—C6—N1	−177.28 (19)	C19—N4—C20—O2	−2.2 (4)
N3—C1—C6—N1	2.2 (3)	C19—N4—C20—C21	177.61 (19)
C7—N1—C6—C5	84.1 (3)	O2—C20—C21—C30	−4.2 (3)
C7—N1—C6—C1	−98.8 (3)	N4—C20—C21—C30	175.96 (18)
C6—N1—C7—N2	173.5 (2)	O2—C20—C21—C22	175.2 (2)
C6—N1—C7—S1	−6.2 (3)	N4—C20—C21—C22	−4.7 (3)
C8—N2—C7—N1	1.5 (3)	C30—C21—C22—C23	1.0 (3)
C8—N2—C7—S1	−178.83 (18)	C20—C21—C22—C23	−178.31 (18)
C7—N2—C8—O1	11.1 (4)	C21—C22—C23—C24	0.5 (3)
C7—N2—C8—C9	−169.4 (2)	C22—C23—C24—C25	176.6 (2)
O1—C8—C9—C10	−146.3 (2)	C22—C23—C24—C29	−1.9 (3)
N2—C8—C9—C10	34.2 (3)	C23—C24—C25—C26	−178.0 (2)
O1—C8—C9—C18	27.9 (3)	C29—C24—C25—C26	0.4 (3)
N2—C8—C9—C18	−151.6 (2)	C24—C25—C26—C27	−1.6 (3)
C18—C9—C10—C11	−1.3 (4)	C25—C26—C27—C28	0.5 (3)
C8—C9—C10—C11	172.9 (2)	C26—C27—C28—C29	1.8 (3)
C9—C10—C11—C16	−1.5 (4)	C27—C28—C29—C30	175.7 (2)
C9—C10—C11—C12	−178.3 (2)	C27—C28—C29—C24	−3.0 (3)
C10—C11—C12—C13	173.7 (2)	C23—C24—C29—C28	−179.69 (19)
C16—C11—C12—C13	−3.1 (4)	C25—C24—C29—C28	1.8 (3)
C11—C12—C13—C14	1.4 (4)	C23—C24—C29—C30	1.6 (3)
C12—C13—C14—C15	0.9 (4)	C25—C24—C29—C30	−176.88 (19)
C13—C14—C15—C16	−1.5 (4)	C22—C21—C30—C29	−1.3 (3)
C14—C15—C16—C11	−0.3 (4)	C20—C21—C30—C29	178.12 (18)
C14—C15—C16—C17	−178.6 (2)	C28—C29—C30—C21	−178.7 (2)
C10—C11—C16—C15	−174.4 (2)	C24—C29—C30—C21	−0.1 (3)
C12—C11—C16—C15	2.5 (4)		

### Hydrogen-bond geometry (Å, °)

**Table:**

*D*—H···*A*	*D*—H	H···*A*	*D*···*A*	*D*—H···*A*
N1—H1···O1	0.89 (2)	1.88 (3)	2.624 (3)	140 (2)
N2—H2A···S1^i^	0.82 (3)	2.60 (3)	3.418 (2)	178 (2)
N3—H3A···O2	0.83 (3)	1.88 (3)	2.613 (3)	148 (3)
N3—H3A···N1	0.83 (3)	2.28 (3)	2.693 (3)	111 (2)
C28—H28···Cg3	0.95	2.76	3.621 (2)	152 (2)

Symmetry codes: (i) −*x*, −*y*+2, −*z*+2.
